# Cost-Effectiveness of Newborn Screening for Infantile-Onset Pompe Disease in Japan

**DOI:** 10.3390/ijns12020021

**Published:** 2026-03-31

**Authors:** Keiko Konomura, Motoko Tanaka, Go Tajima, Eri Hoshino

**Affiliations:** 1 Center for Outcomes Research and Economic Evaluation for Health (C2H), National Institute of Public Health, 2-3-6 Minami, Wako 351-0197, Japan; 2 Division of Neonatal Screening, Research Institute, National Center for Child Health and Development, 2-10-1 Okura, Setagaya 157-8535, Japan; tajima-g@ncchd.go.jp; 3 Division of Policy Evaluation, Department of Health Policy, Research Institute, National Center for Child Health and Development, 2-10-1 Okura, Setagaya 157-8535, Japan; hoshino-e@ncchd.go.jp

**Keywords:** newborn screening, Pompe disease, IOPD, cost-effectiveness analysis, CEA

## Abstract

We conducted a cost-effectiveness analysis of a universal newborn screening (NBS) program for infantile-onset Pompe disease (IOPD) compared with clinical identification in newborns. The analytical model combined a decision tree and a Markov model. The incremental cost-effectiveness ratio (ICER) was estimated over a lifetime horizon, applying a 2% annual discount rate from the public healthcare payer’s perspective. In a cohort of 727,288 individuals, 2.4 patients were expected to have IOPD. The cumulative quality-adjusted life years (QALYs) gained per patient were estimated to be 7.9 when clinically diagnosed and treated with enzyme replacement therapy, and 28.9 when identified through universal NBS. The ICER was 174 million JPY per QALY. Sensitivity and scenario analyses indicated that the parameters most affecting the ICER were the NBS test cost, the quality-of-life value for ambulatory patients, the prevalence of IOPD, and the cost of enzyme replacement therapy. Although considerable uncertainty exists in the analysis, the findings suggest that implementing NBS solely for detecting infantile-onset cases poses challenges in terms of cost-effectiveness, primarily due to the rarity of the disease and the high costs associated with testing and treatment.

## 1. Introduction

Pompe disease is a rare autosomal recessive genetic disorder caused by a congenital deficiency of the enzyme acid alpha-glucosidase, which is responsible for the breakdown of glycogen in lysosomes. This enzymatic deficiency leads to various clinical manifestations involving the cardiovascular, musculoskeletal, respiratory, and gastrointestinal systems. The prevalence of Pompe disease in Japan is estimated to be approximately 1 in 100,000 to 200,000 individuals [1]. Pompe disease is typically classified into infantile-onset and late-onset forms based on age at onset and disease severity [2]. The classic infantile-onset Pompe disease (IOPD) manifests within the first year of life and may be accompanied by symptoms such as hypotonia, respiratory distress, or feeding difficulties. Due to the rapid progression of severe muscle weakness, infants with IOPD often fail to achieve major motor milestones, such as sitting, crawling, or walking. Without treatment, most patients die of cardiorespiratory failure within the first year of life [3]. Late-onset Pompe disease (LOPD) can occur at any age, most commonly after the first year of life, either in childhood or adulthood [4]. LOPD exhibits a wide clinical spectrum; it typically presents with progressive motor disability but rarely involves cardiac manifestations or respiratory failure.

Enzyme replacement therapy (ERT) is available for the treatment of Pompe disease and has been shown to provide therapeutic benefits in both infantile-onset and late-onset forms of the disease [2,5,6,7,8,9,10]. Early initiation of ERT in patients with IOPD has been reported to improve respiratory function and motor outcomes [11].

Newborn screening (NBS) programs have been implemented in several countries [12,13,14,15,16]. The primary goal of NBS is to enable early treatment for patients with IOPD by improving cardiorespiratory function, preserving motor milestones, preventing the need for ventilator support, and reducing mortality [12]. The method used for newborn screening for Pompe disease involves measuring acid alpha-glucosidase activity in dried blood spot samples [2]. The universal NBS program in Japan is a public health initiative targeting all newborns within the first few days after birth. The NBS program was first introduced in Japan in 1977, initially targeting five disorders. In 2014, the adoption of tandem mass spectrometry (MS/MS) led to a significant expansion of the program to include 19 disorders. At present, the NBS program covers 20 disorders following the addition of one more condition [17]. Currently, optional screening for Pompe disease is conducted in certain regions of Japan. In this context, discussions have begun regarding the further expansion of target conditions. To support these discussions, it is essential to evaluate the cost-effectiveness of the NBS program for Pompe disease.

Several previous studies have reported cost-effectiveness analyses of Pompe disease. ERT has been shown to increase the life expectancy and quality of life (QOL) of patients with IOPD compared with conventional treatment. However, it was not considered cost-effective [18,19,20]. Richardson et al. investigated the health and economic outcomes of a universal NBS program for IOPD compared with clinical identification in the United States [21]. Universal NBS screening for Pompe disease led to considerable health gains for patients with IOPD but required substantially higher costs. A cost-effectiveness analysis of a universal NBS program for Pompe disease in the Japanese healthcare context has not yet been conducted. Therefore, this study aims to evaluate the cost-effectiveness of an NBS program for IOPD compared with clinical identification in newborns.

## 2. Materials and Methods

The study was conducted in accordance with the Declaration of Helsinki and approved by the Institutional Review Board of the National Institute of Public Health, Japan (NIPH-IBRA#12424-3, 9 April 2024). The target cohort was defined as all newborns in Japan (*n* = 727,288), based on the 2023 Vital Statistics [22]. The analysis was conducted from the public healthcare payer’s perspective over a lifetime horizon, applying a 2% annual discount rate for both costs and benefits. The analysis was performed using TreeAge Pro 2020 (TreeAge Software, Williamstown, MA, USA). Table 1 presents a summary of the analytical framework. In conducting the cost-effectiveness analysis, we compared outcomes among two strategies: (1) the NBS strategy for IOPD with ERT (NBS strategy) and (2) clinical identification of IOPD with ERT (No NBS strategy).

### 2.1. Model Structure

**Figure 1:**
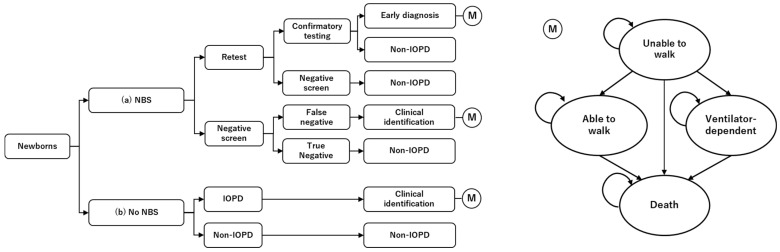
Model structure. NBS: newborn screening; IOPD: infantile-onset Pompe disease. (a) NBS strategy, in which all patients were identified through newborn screening and received enzyme replacement therapy (ERT). (b) No NBS strategy, in which all patients were clinically identified and received ERT. Patients with IOPD were identified using a decision tree model, after which long-term costs and outcomes were estimated using a Markov model. ERT was initiated from the first cycle of the Markov model; however, in the No NBS strategy, treatment initiation was assumed to be delayed by 1.5 months.

The analytical model was developed by combining a decision tree and a Markov model (Figure 1). The decision tree was used to determine the presence or absence of IOPD and the timing of diagnosis through screening, followed by a Markov model to simulate the long-term, disease-specific prognosis. The decision tree model classified individuals into three groups: those with IOPD detected early through screening, those with IOPD identified clinically, and those without IOPD. In the NBS strategy, we assumed that the NBS was performed promptly after birth. In the current NBS program in Japan, blood samples are collected within 4–6 days after birth, and results are returned from the testing facility within one week.

Following NBS, positive cases undergo re-testing using dried blood spot samples; if results remain positive, further confirmatory tests are performed. We assumed that confirmatory testing would detect all true-positive cases and that patients would initiate treatment immediately after a definitive diagnosis. Because Japan has a universal health insurance system and provides financial assistance for children’s medical care, patients are able to receive treatment without significant financial burden. It was assumed that treatment initiation among patients with IOPD would differ by 1.5 months between the NBS and No NBS strategies [12].

We developed a Markov model with a 6-month cycle length comprising four health states: unable to walk, able to walk independently, requiring mechanical ventilation, and death. Although IOPD manifests with various symptoms, defining all relevant health conditions was difficult due to limited evidence. Therefore, the four health states were selected based on data availability and their presumed impact on QOL. This model structure represents that early diagnosis improves ambulation, decreases the need for mechanical ventilation, and reduces mortality. The model was newly developed and underwent face validation by clinical and health economic experts.

### 2.2. Parameters

Table 2 summarizes the parameters used in the analysis. No appropriate studies were identified that directly examined the effectiveness of an NBS program for Pompe disease compared with controls. A single-group large-scale screening study conducted in Japan between April 2013 and October 2020, targeting 297,387 newborns, provided data on the prevalence of IOPD and test performance [23].

Data on patients’ acquisition of walking ability were derived from the results of a large-scale NBS study targeting Pompe disease conducted in Taiwan [24,25]. According to Chien et al. (2015), 80% of patients diagnosed through NBS and treated with ERT achieved independent walking between 14 and 18 months of age, and the remaining patients achieved independent walking by 20 months of age [24]. Therefore, we assumed that all patients identified through NBS and treated with ERT would achieve independent walking between 1.5 and 2 years of age. According to Chien et al. (2009), among patients receiving ERT without NBS, approximately 40% achieved independent walking between 15 and 26 months of age [25]. Therefore, we assumed that 40% of patients receiving ERT without NBS would achieve independent walking between 1 and 2.5 years of age.

**Table 2:** Model parameters.

Variable		Base Case	Range	Reference
Number of births (*n*)		727,288		[22]
Prevalence (*n*)		One in 297,387	(N/A—One in 100,000)	[23]
Sensitivity		1	-	[23]
Specificity		0.9989	-	[23]
Retest rate, %		0.11	(0.085–0.128)	[23]
Proportion receiving confirmatory testing, %		48.7	(39.0–58.5)	[23]
Cost (JPY)	Screening test	3000	(0–5000)	Expert opinion
	Genetic test	38,800	(31,040–46,560)	[26]
	Management cost per month	347,301	(277,841–416,761)	Administrative database
	Ventilation cost per month	102,800	(82,240–123,360)	Administrative database
	Avalglucosidase alfa, 100 mg/vial	196,940	±20%	[26]
Utility	Unable to walk	0.55	(0.40–0.70)	[27]
	Able to walk	0.75	(0.60–0.90)	[27]
	Ventilator-dependent	0.23	(0.08–0.38)	[27]
	General population	0.95	-	[28]

Abbreviations: JPY, Japanese yen.

### 2.3. Cost Data

We included the costs of screening tests and medical care (both inpatient and outpatient) that were relevant from the public healthcare payer’s perspective, with all costs expressed in 2025 prices. Because the exact cost of screening tests under the NBS program was unavailable, we assumed a value of 3000 JPY based on expert opinion. However, since the amount may vary by municipality, its impact was evaluated through sensitivity analysis. The cost of confirmatory testing for lysosomal storage disorder gene analysis was set at 38,800 JPY according to the Medical Fee Points list. Medical expenses were estimated using commercial claims data from the JMDC Inc. (Tokyo, Japan), based on monthly healthcare costs for patients with Pompe disease who had a history of ERT prescriptions [29]. Regarding ventilator costs, since patients requiring ventilatory support are expected to need long-term care, we assumed that positive-pressure ventilators would be used in home care settings. In Japan, both avalglucosidase alfa and alglucosidase alfa are available for treatment. However, according to JMDC data, patients in Japan are predominantly treated with avalglucosidase alfa; therefore, all patients were assumed to have been treated with avalglucosidase alfa. Pharmaceutical costs were calculated based on the Japanese drug price list and standard body weight data derived from the National Health and Nutrition Survey (see Supplementary Table S1) [26,30].

### 2.4. Outcome

No comparative studies on survival outcomes based on NBS programs have been reported in Japan. Therefore, clinical outcomes were informed by the study of Chen et al. (2015) [24], which reported Kaplan–Meier curves for mechanical ventilation–free survival and overall survival in patients identified through NBS and treated with ERT and in clinically identified patients treated with ERT. The curves were digitized using WebPlotDigitizer (version 4.7) [31]. Details of these ventilation–free survival and overall survival rates are presented in Supplementary Figure S1.

According to Chen et al. (2015), no deaths or initiation of mechanical ventilation was observed during the approximately 150-month follow-up period among patients identified through NBS and treated with ERT [24]. Therefore, for patients identified through NBS and treated with ERT, we assumed that mechanical ventilation did not occur and applied general population mortality rates [32].

For clinically identified patients who received ERT, mortality estimates derived from the Kaplan–Meier curves reported by Chien et al. (2015) were applied for the first 48 months [24]. Beyond this period, mortality risk was assumed to stabilize at the Kaplan–Meier plateau, and age-matched general population mortality was applied from 48 to 138 months. After 138 months, patients who achieved ambulation were assumed to experience a reduced mortality risk, informed by clinical evidence indicating functional improvement with ERT in treatment-responsive patients, despite limited direct evidence on long-term survival [33]. In the absence of long-term disease-specific mortality data, these patients were subsequently assumed to follow general population mortality.

For patients in the ventilator-dependent health state, long-term survival data from a cohort of patients with Duchenne muscular dystrophy receiving prolonged ventilatory support were used as a proxy, reflecting the shared pathophysiology of progressive respiratory muscle weakness [34]. A hazard rate of 0.0257 was applied as a constant excess disease-specific mortality under an exponential survival assumption. Due to the absence of state-specific survival data, non-ambulatory and ventilator-dependent patients were modelled as a single advanced disease state, to which a common overall survival function was applied to derive mortality transition probabilities.

A literature review was conducted to identify QOL values; however, no appropriate QOL studies for IOPD were found. Therefore, QOL values from a clinical trial of ERT for LOPD were adopted [27]. Health state utility values were 0.55 (unable to walk), 0.75 (able to walk), and 0.23 (ventilator-dependent). Age-related changes in QOL values were estimated using age-specific adjustment factors derived from Japanese population norm values [28]. The age group of 16–19 years was used as the reference, and adjustment factors were calculated as the ratio of the population norm value at each age during the analysis to that of the reference group. For individuals aged 90 years and older, QOL values were assumed to be the same as those for the 80–89-year age group.

### 2.5. Analysis Plan

Effectiveness was measured in life-years (LYs) and quality-adjusted life years (QALYs), and the incremental cost-effectiveness ratio (ICER) was calculated in JPY per LY and per QALY. The base-case analysis was conducted over a lifetime horizon using the most plausible parameter values. A one-way sensitivity analysis was performed to assess model uncertainty. For parameters with plausible ranges reported in the literature or obtained from expert opinion, those ranges were applied. For parameters without established ranges, values were varied by ±20% around their base-case estimates. Given the inherent uncertainty of long-term projections, a scenario analysis was performed using a 20-year time horizon.

## 3. Results

There were 2.4 cases of IOPD in the cohort of 727,288 newborns. Lifetime QALYs and costs among patients with IOPD were estimated to be 7.9 and 1.54 billion JPY in the No NBS strategy, and 28.9 and 4.31 billion JPY under early detection via NBS. In the NBS strategy, lifetime total costs were 12.74 billion JPY, of which drug costs accounted for 79.5% (10.13 billion JPY) and NBS screening costs for 17.1% (2.18 billion JPY). In the No NBS strategy, lifetime total costs were 3.78 billion JPY, of which drug costs accounted for 95.8% (3.62 billion JPY). The results of the base-case and scenario analyses are presented in Table 3. The incremental LYs estimated over the lifetime horizon were 0.000079 between the NBS and No NBS strategies. The incremental cost of the NBS strategy, relative to the No NBS strategy, was estimated at 12,317 JPY, with an incremental effect of 0.000071 QALYs. This resulted in an ICER of 174,159,534 JPY per QALY gained.

**Figure 2:**
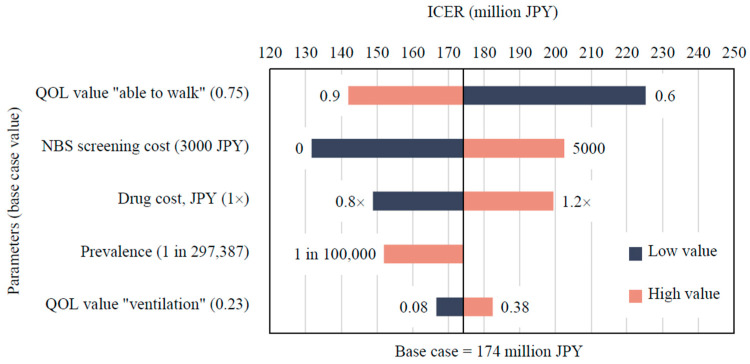
Tornado diagram comparing the NBS and No NBS strategies. ICER, incremental cost-effectiveness ratio; NBS, newborn screening; JPY, Japanese yen; QOL, quality of life.

One-way sensitivity analysis identified the key parameters influencing the ICER as follows: drug cost, prevalence rate, NBS screening cost, and utility value for the “able to walk” health state (Figure 2). When the prevalence increased to approximately one in 100,000 live births, the ICER decreased to 152 million JPY per QALY. If the NBS screening cost was hypothetically reduced to zero, the ICER declined to 132 million JPY per QALY.

## 4. Discussion

The NBS program aimed at the early detection of IOPD in newborns was found to pose economic challenges compared with clinical identification. However, NBS improved the QALYs of patients with IOPD. The estimated lifetime QALYs were 7.9 for the No NBS strategy and 28.9 for the NBS strategy. Although the NBS intervention resulted in an approximate gain of 21 QALYs per patient, the overall incremental QALYs were limited because of the very low disease prevalence. In the base-case analysis, the prevalence of IOPD was assumed to be approximately one in 300,000 live births. In the sensitivity analysis, even when the prevalence increased to one in 100,000, the ICER remained high at 152 million JPY per QALY. In Japan, the commonly accepted willingness-to-pay (WTP) threshold is approximately 5 to 6 million JPY per QALY [35,36], and our analysis yielded values far exceeding this range.

A cost-effectiveness evaluation of NBS for patients with IOPD has been reported in the United States [21]. That study estimated the health and economic outcomes associated with universal NBS for IOPD compared with clinical identification, assuming that all confirmed cases received alglucosidase alfa. From a health sector perspective in the United States, the ICER was estimated at USD 408,000 per QALY gained, which far exceeds the commonly accepted cost-effectiveness threshold in that setting [37]. Our findings are generally consistent with those reported in that study.

Most of the total costs in both the NBS and No NBS strategies were driven by drug costs and NBS screening costs. The NBS screening cost, though relatively small, is applied to the entire newborn population, whereas the drug—although administered to only a small number of patients—is extremely expensive. Consequently, these two components had a major influence on the ICER. Even when the NBS screening cost was assumed to be 0 JPY, the ICER remained high, exceeding 155 million JPY per QALY. Sensitivity and scenario analyses also indicated that drug costs substantially affect the ICER; however, the ICER remained high despite these variations. These findings suggest that even if the NBS screening cost were reduced, improvements in cost-effectiveness would remain limited as long as the treatment remains highly expensive. The high drug cost is largely attributable to the substantial financial investment required for the development of therapies for rare diseases. This issue similarly applies to other disorders recently considered for inclusion in NBS panels, for which new high-cost therapies have also been introduced.

Because NBS is funded by public resources, transparent discussion is necessary to justify its implementation. In Japan, there is ongoing debate about expanding NBS; however, the criteria for selecting target conditions have not been explicitly defined. We previously proposed a set of evaluation items that should be considered when selecting conditions [38]. Economic evaluation is one of these key components, and the results of this study contribute to the evidence to inform that aspect of the decision-making process, including by clarifying the uncertainties involved.

This analysis focused exclusively on the early detection of IOPD, which directly reflects the primary objective of NBS implementation in Japan. Implementing an NBS program may also identify additional cases of LOPD in individuals who are asymptomatic at diagnosis. This may lead to additional costs associated with long-term monitoring and follow-up until symptom onset, as well as an increased number of patients initiating ERT. However, because of the broad clinical spectrum of LOPD and the limited knowledge of its long-term prognosis, LOPD cases were not included in this analysis.

The primary limitation of this study was the uncertainty in the analysis arising from the limited availability of data. First, the clinical outcomes associated with both the implementation of NBS and the earlier initiation of treatment were derived from unadjusted results reported in an observational study conducted in Taiwan [11]. Although that study provides valuable insights with an observation period exceeding 10 years, potential biases due to differences in measurement time points among patient groups and the small sample size cannot be excluded. Nevertheless, such issues are inherent limitations commonly observed in studies of rare diseases.

Second, regarding QOL values, no domestic data were available, and only findings from international studies of LOPD could be used [27]. Considering potential differences in health-related QOL perceptions across countries, and recognizing that utility values can influence model outcomes, it would be preferable to use QOL data derived from a Japanese population. In particular, the measurement and valuation of QOL in pediatric and rare disease populations remain globally recognized methodological challenges. To enable more precise evaluations of diseases targeted by NBS programs, addressing these issues should be a priority for future research.

In this study, we did not account for differences in management costs according to walking ability. In general, costs may increase as walking ability declines due to disease progression and increasing severity; however, such information was not available in the dataset used for cost estimation. Failure to account for these costs may have led to an underestimation of costs in the No NBS strategy. Nevertheless, because drug costs account for the majority of total costs, the impact of this omission is likely to be limited.

Due to limited data availability, probabilistic sensitivity analysis could not be conducted. Instead, deterministic sensitivity and scenario analyses were performed. The data limitations contributed substantially to the uncertainty of the analysis, and the sensitivity analyses demonstrated that the ICER was highly responsive to parameter variation. Nevertheless, the ICER remained consistently high across all changes.

## 5. Conclusions

This study conducted a cost-effectiveness analysis of implementing an NBS program for the detection of IOPD in the Japanese newborn population. When focusing solely on IOPD, the analysis indicated limited cost-effectiveness, primarily due to the rarity of the disease and the relatively high combined costs of screening and treatment. However, the currently available evidence regarding the clinical utility of NBS implementation and the epidemiology of Pompe disease in Japan remains limited. To enhance the precision and robustness of future economic evaluations, further data collection in these areas will be essential.

## Figures and Tables

**Table 1:** Summary of the analytical framework.

Item	Description
Population	All newborns in Japan
Intervention	Population-based newborn screening strategy for infantile-onset Pompe disease
Comparator	Clinical identification
Cost perspective	Public healthcare payer’s perspective
Time horizon	Lifetime from birth
Study design	Model-based (decision tree model with an embedded Markov model)
Discount rate	2.0% per year
Outcomes	Life-years, quality-adjusted life years, and incremental cost-effectiveness ratio

**Table 3:** Results of Cost-effectiveness Analysis.

Strategy		Total QALYs	Incremental QALYs	Total LYs	Incremental LYs	Cost per Infant (JPY)	Incremental Cost (JPY)	ICER (JPY per LY)	ICER (JPY per QALY)
Lifetime									
	NBS	37.296969	0.000071	40.522141	0.000079	17,518	12,317	155,723,889	174,159,534
	No NBS	37.296898	-	40.522062	-	5201	-	-	-
20 years									
	NBS	15.610370	0.000026	16.468772	0.000024	7034	5011	208,602,824	195,274,920
	No NBS	15.610345	-	16.468747	-	2023	-	-	-

Abbreviations: ICER, incremental cost-effectiveness ratio; NBS, newborn screening; QALY, quality-adjusted life-year; LY, life-year.

## Data Availability

The original data presented in this study are included in the article. Further inquiries can be directed to the corresponding author.

## Supplementary Materials

The following supporting information can be downloaded at: https://www.mdpi.com/article/10.3390/ijns12020021/s1, Figure S1: Survival curves used in the economic model; Table S1: Cost of avalglucosidase alfa.

## Abbreviations

The following abbreviations are used in this manuscript:

ERT	Enzyme replacement therapy
ICER	Incremental cost-effectiveness ratio
IOPD	Infantile-onset Pompe disease
JPY	Japanese yen
LY	Life-year
NBS	Newborn screening
QALY	Quality-adjusted life-year
QOL	Quality of life
