# Supplementary material for: Cost-Effectiveness of Newborn Screening for Infantile-Onset Pompe Disease in Japan
Source: Int J Neonatal Screen. 2026 Mar 31;12(2):21. doi: 10.3390/ijns12020021 (PMC13108023; doi:10.3390/ijns12020021)
Supplement: Supplementary file 1 [file IJNS-12-00021-s001.zip › IJNS-4026868-supplementary.pdf]

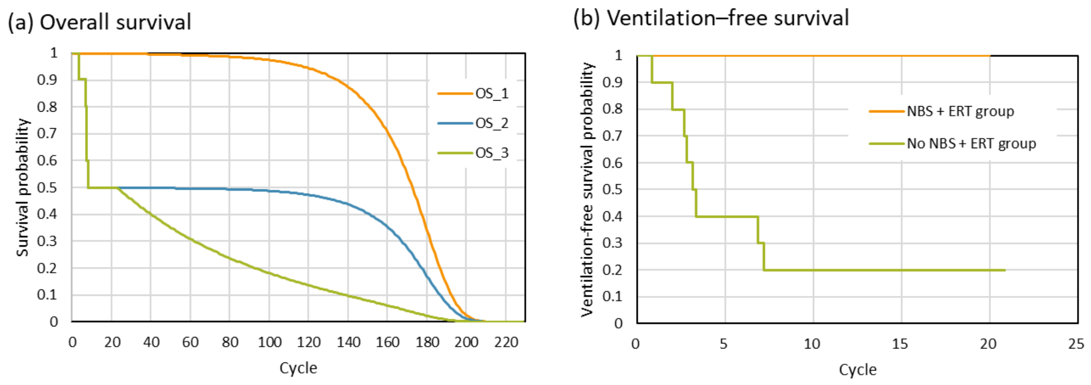


**Figure S1. Survival Curves Used in the Economic Model.**

The cycle length was six months. Panel (a) illustrates the overall survival curves applied to each health state. The OS_1 curve was applied to both the “unable to walk” and “able to walk” health states in the NBS strategy. The OS_2 curve was applied to the “able to walk” health state in the No NBS strategy. The OS_3 curve was applied to the “unable to walk” and “ventilator-dependent” health states in the No NBS strategy. Panel (b) presents the Kaplan–Meier curves for ventilation-free survival.

**Table S1. Cost of avalglucosidase alfa.**

| Age (years) | Base | Low | High | Reference |
|---|---|---|---|---|
| 1 | 20,520,801 | 16,416,641 | 24,624,961 | |
| 2 | 25,161,956 | 20,129,565 | 30,194,348 | |
| 3 | 28,448,041 | 22,758,433 | 34,137,649 | |
| 4 | 33,787,928 | 27,030,343 | 40,545,514 | |
| 5 | 36,851,405 | 29,481,124 | 44,221,686 | |
| 6 | 42,097,216 | 33,677,773 | 50,516,659 | |
| 7 | 47,667,650 | 38,134,120 | 57,201,180 | |
| 8 | 53,093,675 | 42,474,940 | 63,712,410 | |
| 9 | 62,136,150 | 49,708,920 | 74,563,380 | |
| 10 | 67,829,373 | 54,263,499 | 81,395,248 | |
| 11 | 79,755,111 | 63,804,089 | 95,706,133 | |
| 12 | 85,455,426 | 68,364,341 | 102,546,512 | |
| 13 | 96,133,005 | 76,906,404 | 115,359,606 | |
| 14 | 107,090,113 | 85,672,090 | 128,508,136 | |
| 15 | 113,140,218 | 90,512,174 | 135,768,261 | |
| 16 | 112,309,408 | 89,847,526 | 134,771,289 | [26,30] |
| 17 | 119,409,384 | 95,527,507 | 143,291,261 | |
| 18 | 113,447,613 | 90,758,091 | 136,137,136 | |
| 19 | 111,898,647 | 89,518,918 | 134,278,377 | |
| 20 | 108,621,851 | 86,897,481 | 130,346,222 | |
| 21 | 122,319,163 | 97,855,330 | 146,782,996 | |
| 22 | 120,390,345 | 96,312,276 | 144,468,414 | |
| 23 | 126,721,329 | 101,377,064 | 152,065,595 | |
| 24 | 120,411,966 | 96,329,572 | 144,494,359 | |
| 25 | 118,798,986 | 95,039,188 | 142,558,783 | |
| 26-29 | 126,642,284 | 101,313,827 | 151,970,741 | |
| 30-39 | 127,193,061 | 101,754,449 | 152,631,673 | |
| 40-49 | 131,360,288 | 105,088,231 | 157,632,346 | |
| 50-59 | 129,141,303 | 103,313,042 | 154,969,563 | |
| 60-69 | 124,920,197 | 99,936,157 | 149,904,236 | |
| 70+ | 116,228,861 | 92,983,089 | 139,474,633 | |

Because pharmaceutical costs depend on patient body weight, age-specific effects were considered in the analysis. Annual pharmaceutical costs were calculated based on the Japanese drug price list and standard body weight [26] data derived from the National Health and Nutrition Survey [30].
